# The prognostic significance of further axillary dissection for sentinel lymph node micrometastases in female breast cancer: A competing risk analysis using the SEER database

**DOI:** 10.3389/fonc.2022.1012646

**Published:** 2022-11-17

**Authors:** Yudong Zhou, Shengyu Pu, Siyuan Jiang, Danni Li, Shouyu Li, Yang Liu, Yu Ren, Na Hao

**Affiliations:** ^1^ Department of Breast Surgery, the First Affiliated Hospital of Xi’an Jiaotong University, Xi’an, Shaan’xi, China; ^2^ School of Medicine, Xi’an Jiaotong University, Xi’an, Shaan’xi, China

**Keywords:** micrometastasis, sentinel lymph node, axillary lymph node dissection, breast cancer, SEER (surveillance epidemiology and end results) database

## Abstract

**Background:**

Sentinel lymph node (SLN) biopsy has been widely recognized as an excellent surgical and staging procedure for early-stage breast cancer, and its development has greatly improved the detection of micrometastases. However, the axillary treatment of micrometastasis has been the subject of much debate.

**Methods:**

We identified 427,131 women diagnosed with breast cancer from 2010 to 2018 in the Surveillance, Epidemiology, and End Results (SEER) database. Patients whose nodal status was micrometastases (pTxN1miM0) were classified into two groups: the SLNB only group and SLNB with complete ALND group, and we used these classifications to carry out propensity-score matching (PSM) analysis. The primary and secondary endpoints were OS and BCSS, respectively. We then implemented the Kaplan-Meier method and Cox proportional hazard model and used Fine and Gray competitive risk regression to identify factors associated with the risk of all-cause mortality.

**Results:**

After the PSM, 1,833 pairs were included in total. The SLNB with complete ALND showed no significant difference in OS (HR=1.04, 95% CI: 0.84-1.28, P=0.73) or BCSS (HR= 1.03, 95% CI: 0.79-1.35, P=0.82) compared to the SLNB only group, and axillary treatment was not associated with breast cancer-specific death (BCSD) (HR=1.13, 95% CI: 0.86-1.48, P=0.400) or other cause-specific death (OCSD) (HR=0.98, 95% CI:0.70-1.38, P=0.920). There was no statistically significant difference in the cumulative incidence of BCSD (Grey’s test, P=0.819) or OCSD (Grey’s test, P=0.788) for between the two groups either. For different molecular subtypes, patients in the SLNB only group showed no statistically significant differences from those in the SLNB with complete ALND group with Luminal A (HR=1.00, 95% CI:0.76-1.32, P=0.98) or Luminal B (HR=0.82, 95% CI:0.42-1.62, P=0.55) but similar OS to HER2-enriched (HR=1.58, 95% CI:0.81-3.07, P=0.19) or triple negative breast cancers (HR=1.18, 95% CI:0.76-1.81, P=0.46).

**Conclusions:**

Our results suggest that in early breast cancer patients with micrometastasis, complete ALND does not seem to be required and that SLNB suffices to control locoregional and distant disease, with no significant adverse effects on survival compared to complete ALND.

## Introduction

Sentinel lymph nodel biopsy (SLNB) is now widely recognized as an excellent surgical and staging procedure for early-stage breast cancer (1–3), and for sentinel lymph node (SLN) metastases breast cancer patients, axillary lymph node dissection (ALND) has remained a complementary treatment for most patients (4). For patients with negative SLN results, the NSABP-B-32 trial suggested that ALND can be omitted and that SLNB can be safely performed with no impact on the overall and disease-free survival and locoregional disease control (5, 6). However, with multi-section and improvements in histopathological and molecular analysis, more and more SLN micrometastases are being detected, which is pathologically staged as stage N1mi and defined as tumor invasion in lymph nodes greater than 0.2mm and/or more than 200 cells but no greater than 2.0mm (7–9).

Numerous studies suggest that nodal micrometastasis is an independent risk factor for breast cancer mortality, however, and should not be considered the same as that in truly node-negative patients (3, 10). In addition, some researchers have suggested that patients with micrometastases without further ALND would not suffer from a high incidence of regional recurrence and questioned the need for ALND in breast cancer patients with SLN micrometastases (3, 9, 11–14). Therefore, researchers have begun to question the need for axillary treatment of certain SLN micrometastases.

Following the NSABP-B-32 trial, the IBCSG 23-01 trial recently provided evidence that SLNB alone, without complete ALND, could be extended to early-stage breast cancer patients presenting only micrometastasis in the sentinel lymph node (15), and the ACOSOG-Z011 trial demonstrated that ALND was probably not necessary for female breast cancer with SLN micrometastases (16–18). An analysis of studies using the National Cancer Data Base (NCDB) and a prospective, randomized clinical trial (AATRM 048/13/2000) also came to the same conclusion that SLNB with complete ALND did not appear to be associated with a significant improvement in survival in SLN micrometastases (19). Recently, the SERC (Sentinelle Envahi et Randomisation du Curage) and the SENOMIC (Sentinelle node Micrometastasis) trials were designed with the intention of confirming the safety of the ALND omission in the populations of patients who were under-represented in previously published trials (20, 21). However, the above cited trials were not designed to specifically evaluate patients presenting SN micro-metastasis specifically. For example, the ACOSOG-Z011 trial did not differentiate patients with micro-metastases from patients with macro-metastases (17), and the IBCSG-23-01 trial did not differentiate between ITC and micro-metastasis (15). Additionally, the AATRM 048/13/2000 trial was designed to only to evaluate patients with SN micro-metastases (19). Both the IBCSG-23-01 and the AATRM 048/13/2000 trials included low numbers of patients who underwent mastectomy: 86 (9.2%) patients and 18 patients, respectively (15, 19). What’s more, their current guidelines recommended that ALND should be completed when lymph node involvement is identified by SLNB (1, 22), while ALND is considered potentially to provide additional prognostic information for breast cancer patients after surgery and possibly to reduce axillary recurrence (23–25). Thus, the question remains whether further ALND is indicated in patients with SLN micro-metastases.

To further explore the prognostic value of SLN micrometastasis further and to identify whether omitting ALND has an impact on breast cancer-specific survival (BCSS) and overall survival (OS) in breast cancer patients with micrometastases, we followed a large cohort of female breast cancer patients with stage pTxN1miM0 from 2010 to 2018 using the population-based database Surveillance, Epidemiology, and End Results (SEER) registry program. We then applied statistical methods, such as the Kaplan-Meier method, Cox proportional hazards model, and competing risk analysis model, were preformed to further analyze the efficiency and prognostic factors of ALND for patients with SLN micrometastases.

## Methods

### Data resources

In this study, we extracted the breast cancer cases from the SEER database, totaling 18 population-based cancers, using the SEER*Stat program version 8.3.9 (https://seer.cancer.gov/seerstat/) (26). The National Cancer Institute’s SEER program collects information on cancer incidence, survival and patient demographics that represent approximately 28% of the US population (27). All procedures were performed in accordance with approved guidelines. As the SEER database is publicly accessible, informed patient consent was not required for this study. Therefore, the research was deemed exempt from review by the Ethics Committee of the First Affiliated Hospital of Xi’an Jiaotong University.

### Patient cohort

**Figure 1:**
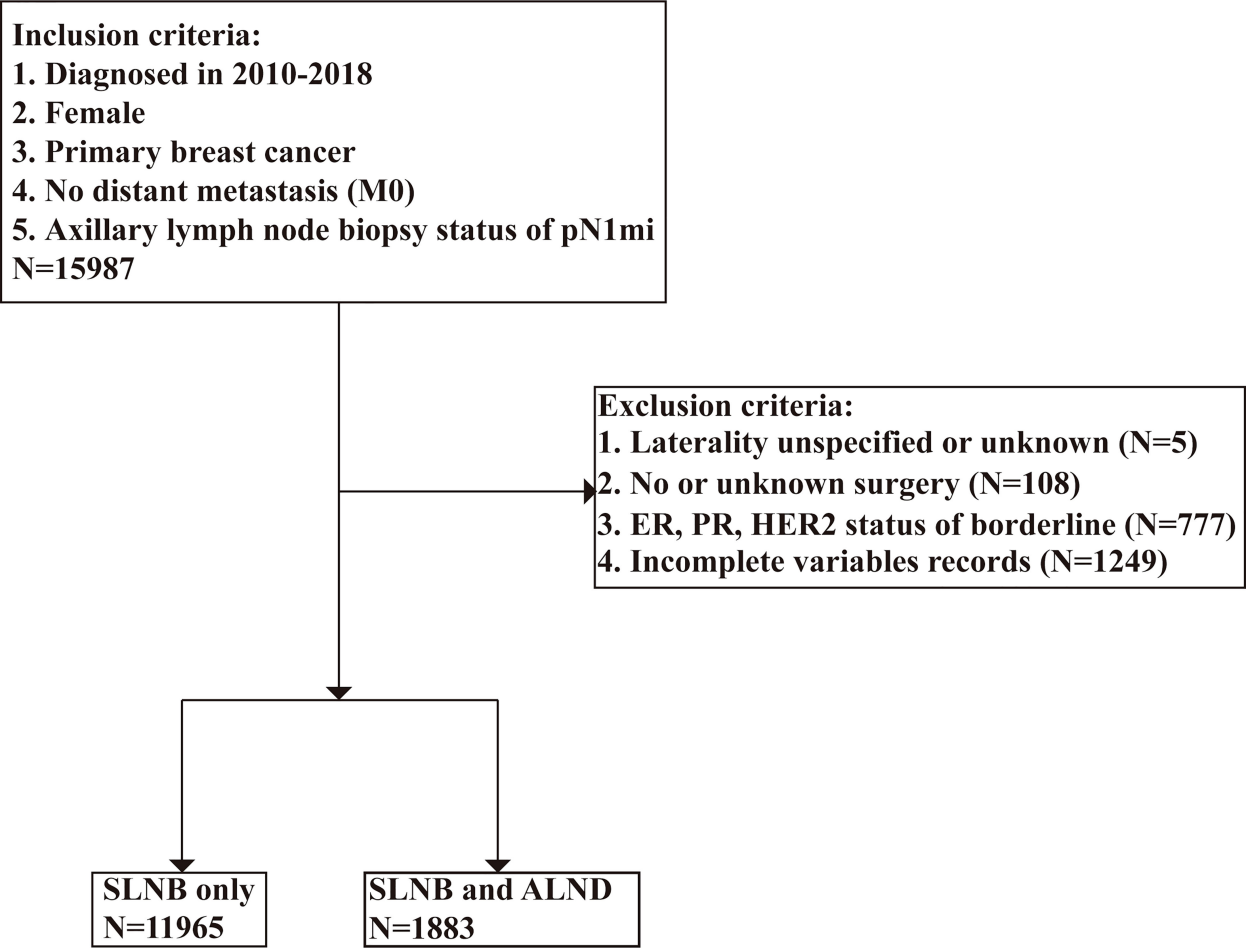
Eligibility, inclusion, and exclusion criteria of the study population.

We used the SEER*Stat program was used to identify 427,131 patients who had received a breast cancer diagnosis from 2010 to 2018. To be enrolled in this research, patients must have had a pathologically-confirmed for lymph node biopsy status of pN1mi breast cancer at any point in the database in one of the 18 SEER-covered registries. The following variables were extracted: survival months, age at diagnosis, marital status, race, grade, laterality, CS tumor size, 7^th^ edition AJCC classification (28), subtype, ER status, PR status, HER2 status, radiation recode, chemotherapy recode, surgery status, lymph node examination, cause of death, and vital status. The inclusion criteria were as follows: [1] female; [2] primary breast cancer; [3] no distant metastasis; and [4] axillary lymph node biopsy status of pN1mi. After the preliminary subject selection, patients were excluded using the following criteria: [1] laterality unspecified or unknown; [2] missing surgery records; [3] ER, PR, or HER2 status of borderline; and [4] incomplete variables records. The selection procedure is shown in **Figure 1**.

In total 13,848 patients with pTxN1miM0 were included in our cohort. To evaluate the effect of axillary lymph node dissection on prognosis, the study cohort was divided into two groups according to the SEER program surgery codes for breast cancer. If a patient underwent local tumor destruction, partial mastectomy, subcutaneous mastectomy, or total mastectomy, the patient was categorized into the SLNB only group. If a patient underwent modified radical mastectomy, radical mastectomy or extended radical mastectomy, the patient was classified into the SLNB with complete ALND group. We considered “no radiation and/or cancer-directed surgery” as no radiotherapy, and “no/unknown” chemotherapy recodes as no chemotherapy.

### Endpoints

Patients were followed up with until November 2018, and the median follow-up time was 48 months (ranging from 0 to 107 months). The primary endpoint was overall survival, defined as the time from the date of diagnosis to death. Secondary outcome measurements were breast cancer-specific survival, breast cancer-specific death (BCSD) and other cause-specific death (OCSD). We defined both BCSS and BCSD were defined as the time interval between the date of diagnosis and death due to breast cancer and OCSD as the time from the date of diagnosis to the date of death from other causes.

## Statistics analysis

The Pearson chi-square test or Fisher’s exact test were utilized as appropriate to examine the differences in patient demographics and clinical variables between groups. Propensity score matching (PSM) was performed to balance differences in the patients clinicopathological factors used between the two groups by 1:1 ratio matching. The R package “MatchIt” was utilized to run the PSM procedure. Survival curves were generated *via* Kaplan-Meier analysis, and log-rank tests were performed to determine statistical differences between groups by using the R packages “survival’’ and “survminer”. We followed this analysis with univariate and multivariate Cox regression analysis in order to investigate the prognostic factors that were independently associated with OS.

Then, to explore the effect of axillary lymph node dissection in different molecular subtypes, we further classified the enrolled population into four subgroups (Luminal A, Luminal B, HER2 enriched, and Triple negative) depending on molecular typing. Differences between patients in the SLNB only and SLNB with complete ALND groups were then examined with the Kaplan-Meier method.

We used competing risk model analysis to separate the causes of death into BCSD and OCSD subgroups to mitigate bias in the estimations (29). In our multivariate survival competitive risk analysis, we used the Fine and Gray competitive risk regression to identify factors associated with the risk of all-cause mortality using the R package “cmprsk”. All statistical analysis was carried out using SPSS version 22.0 (IBM Corporation) and R (version 4.1.3, http://www.R-project.org/). All stated P values are for two-sided tests, and P<0.05 was considered to indicate statistical significance.

## Results

### Baseline characteristics of patients

The baseline clinical characteristics of the included patients are shown in **Table 1**. Of the 13,848 women included in the current study, a total of 11,965 participants were in the SLNB only group, and 1,883 participants were in the SLNB with complete ALND group. Among these women, 9,983 (73.4%) were age 50 or older, 8,615 (62.2%) were married, 10,882 (78.6%) were white, and 6,903 (49.8%) had been diagnosed with left breast cancer. In total, 6,840 (49.4%) patients were moderately differentiated (grade II), 5,012 (36.2%) were at the T2 stage, 10,960 (79.2%) were Luminal A subtype, 1447 (10.4%) were Luminal B subtype, 440 (3.2%) were HER2 enriched subtype, 1001 (7.2%) were Triple negative subtype, 12,309 (88.9%) were ER positive (ER +), 11,106 (80.2%) were PR positive (PR +), and 1,887 (13.6%) were HER2 positive (HER2 +). A total of 8,070 (58.3%) cases received radiotherapy, and 6,906 (49.9%) cases received chemotherapy. By comparing patients in the SLNB only and SLNB with complete ALND groups, we found statistically significant differences (p < 0.05) in the age at diagnosis, marital status, race, grade, T stage, subtype, ER status, PR status, HER2 status, radiation, and chemotherapy treatment subgroups. We then employed PSM to avoid potential prognostic confounders that could affect the accuracy of the results. After PSM, only 3,766 patients were included, with 1,883 patients in the SLNB only group and 1,883 patients in the SLNB with complete ALND group. Here, we observed no differences in terms of the aforementioned covariates. Key methodological characteristics are shown in **Table 1**. To further explore the patients with a single or 2 (macroscopic) metastatic lymph nodes, we screened 1768 pairs of patients by matching in the original data. Key methodological characteristics are shown in **Table S1**.

**Table 1:** Patient clinical and pathological characteristics.

Characteristic		Before PSM				After PSM		
	Total patients	SLNB	SLNB+ALND	p value	Total patients	SLNB	SLNB+ALND	p value
	(n = 13848)	(n = 11965)	(n = 1883)		(n = 3766)	(n = 1883)	(n = 1883)	
Age (mean (SD))	57.95 (12.90)	58.14 (12.74)	56.77 (13.83)	<0.001	56.71 (13.84)	56.64 (13.85)	56.77 (13.83)	0.778
Age (years)								
<50	3685 (26.6)	3252 (27.2)	613 (32.6)	<0.001	1265 (33.6)	652 (34.6)	613 (32.6)	0.178
≥50	9983 (73.4)	8713 (72.8)	1270 (67.4)		2501 (66.4)	1231 (65.4)	1270 (67.4)	
Marital status								
Married	8615 (62.2)	7520 (62.8)	1095 (58.2)	<0.001	2197 (58.3)	1102 (58.5)	1095 (58.2)	0.817
Other	5233 (37.8)	4445 (37.2)	788 (41.8)		1569 (41.7)	781 (41.5)	788 (41.8)	
Race,								
White	10882 (78.6)	9447 (79.0)	1435 (76.2)	0.002	2907 (77.2)	1472 (78.2)	1435 (76.2)	0.351
Black	1519 (11.0)	1269 (10.6)	250 (13.3)		477 (12.7)	227 (12.0)	250 (13.3)	
Other	1447 (10.4)	1249 (10.4)	198 (10.5)		382 (10.1)	184 (9.8)	198 (10.5)	
Grade								
I	2718 (19.6)	2462 (20.6)	256 (13.6)	<0.001	515 (13.7)	259 (13.8)	256 (13.6)	0.851
II	6840 (49.4)	5959 (49.8)	881 (46.8)		1776 (47.1)	895 (47.5)	881 (46.8)	
III and IV	4290 (31.0)	3544 (29.6)	746 (39.6)		1475 (39.2)	729 (38.7)	746 (39.6)	
Laterality								
Left	6903 (49.8)	5944 (49.7)	959 (50.9)	0.313	1896 (50.3)	937 (49.8)	959 (50.9)	0.473
Right	6945 (50.2)	6021 (50.3)	924 (49.1)		1870 (49.7)	946 (50.2)	924 (49.1)	
Tumor size mean (SD)	23.64 (19.10)	22.46 (17.84)	31.17 (24.42)	<0.001	30.52 (21.93)	29.87 (19.11)	31.17 (24.42)	0.068
AJCC T stage								
T0/1	7877 (56.9)	7152 (59.8)	725 (38.5)	<0.001	1465 (38.9)	740 (39.3)	725 (38.5)	0.365
T2	5012 (36.2)	4150 (34.7)	862 (45.8)		1740 (46.2)	878 (46.6)	862 (45.8)	
T3/4	959 (6.9)	663 (5.5)	296 (15.7)		561 (14.9)	265 (14.1)	296 (15.7)	
Subtype								
Luminal A	10960 (79.2)	9605 (80.3)	1355 (71.9)	<0.001	2732 (72.6)	1377 (73.1)	1355 (71.9)	0.807
Luminal B	1447 (10.4)	1205 (10.1)	242 (12.9)		465 (12.3)	223 (11.9)	242 (12.9)	
HER2 enriched	440 (3.2)	348 (2.9)	92 (4.9)		184 (4.9)	92 (4.9)	92 (4.9)	
Triple negative	1001 (7.2)	807 (6.7)	194 (10.3)		385 (10.2)	191 (10.1)	194 (10.3)	
ER Status								
Positive	12309 (88.9)	10734 (89.7)	1575 (83.6)	<0.001	3166 (84.1)	1591 (84.5)	1575 (83.6)	0.476
Negative	1539 (11.1)	1231 (10.3)	308 (16.4)		600 (15.9)	292 (15.5)	308 (16.4)	
PR Status								
Positive	11106 (80.2)	9740 (81.4)	1366 (72.5)	<0.001	2756 (73.2)	1390 (73.8)	1366 (72.5)	0.377
Negative	2742 (19.8)	2225 (18.6)	517 (27.5)		1010 (26.8)	493 (46.2)	517 (27.5)	
HER2 Status								
Positive	1887 (13.6)	1553 (13.0)	334 (17.7)	<0.001	649 (17.2)	315 (16.7)	334 (17.7)	0.412
Negative	11961 (86.4)	10412 (87.0)	1549 (82.3)		3117 (82.8)	1568 (83.3)	1549 (82.3)	
Radiation								
Yes	8070 (58.3)	7449 (62.3)	621 (33.0)	<0.001	1217 (32.3)	596 (31.7)	621 (33.0)	0.384
No/unknown	5778 (41.7)	4516 (37.7)	1262 (67.0)		2549 (67.7)	1287 (68.3)	1262 (67.0)	
Chemotherapy								
Yes	6906 (49.9)	5736 (47.9)	1170 (62.1)	<0.001	2352 (62.5)	1182 (62.8)	1170 (62.1)	0.686
No/unknown	6942 (50.1)	6229 (52.1)	713 (37.9)		1414 (37.5)	701 (37.2)	713 (37.9)	

SLNB, sentinel lymph node biopsy; ALND, axillary lymph node dissection; PSM, propensity-score matching; SD, standard deviation; ER, estrogen receptor; PR, progesterone receptor; HER2, human epidermal growth factor receptor 2.

### Kaplan-meier survival analysis

**Figure 2:**
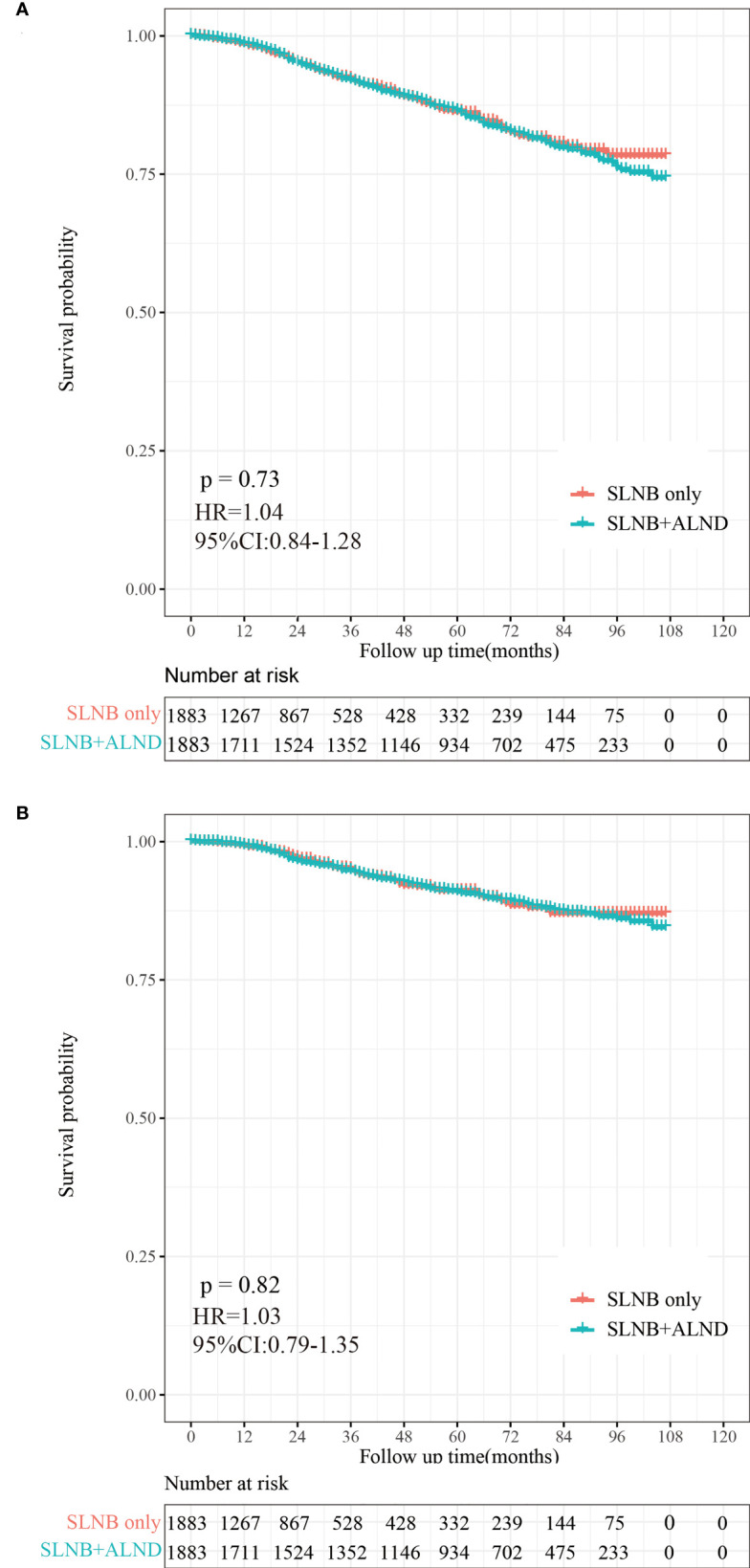
Kaplan-Meier survival analysis for pTxN1miM0 female breast cancer patients after PSM. **(A)** Overall survival curves in the SLNB only group and SLNB with complete ALND group. **(B)** Breast cancer-specific survival curves in the SLNB group and SLNB with complete ALND group.

A total of 406 (10.78%) patients died in this cohort study, and 60.59% (246/406) of them had a breast cancer-specific death. The OS after three, five, and eight years was 92.26%, 86.26%, and 78.34% in the SLNB only group, respectively, and 91.96%, 86.43%, and 76.08% in the SLNB with complete ALND group, respectively (**Figure 2A**). The BCSS after three, five, and eight years was 94.99%, 91.08%, and 86.98% in the SLNB only group, respectively, and 94.71%, 90.86%, and 86.05% in the SLNB with complete ALND group, respectively (**Figure 2B**). The hazard ratio (HR) demonstrates the risk of OS and BCSS. As shown in **Figures 2A, B**, the SLNB with complete ALND group showed had no significant difference in OS (HR=1.04, 95% CI: 0.84-1.28, P=0.73) or BCSS (HR= 1.03, 95% CI: 0.79-1.35, P=0.82) compared to the SLNB only group. As for the patients with a single or 2 (macroscopic) metastatic lymph nodes, the SLNB with complete ALND group showed had no significant difference in OS (HR=1.06, 95% CI: 0.86-1.31, P=0.80) or BCSS (HR= 1.03, 95% CI: 0.79-1.35, P=0.58) compared to the SLNB only group (**Figures S1A, B**).

### Univariate and multivariate cox regression analysis

The results of our univariate Cox analysis showed that the axillary treatment was not associated with improved OS (P= 0.728) or BCSS (P= 0.820) (**Table 2**). In addition, laterality, HER2 status, and radiation were also not related to the OS or BCSS (all P>0.05). Furthermore, the univariate analysis also showed that age, marital status, grade, T stage, subtype, ER, and PR status were significantly associated with OS and BCSS (all P<0.05) (**Table 2**). Moreover, our univariate analysis showed that patients who did not receive chemotherapy were statistically significantly associated with shorter OS (HR=1.50, 95% CI: 1.23-1.82, P<0.001) but not with BCSS (HR=0.81, 95% CI: 0.61-1.07, P=0.130). As shown in **Table S2**, similar results were archived in the cohort of the patients with a single or 2 (macroscopic) metastatic lymph nodes.

**Table 2:** Univariate cox regression model analysis.

characteristic	OS		BCSS	
	HR[95% CI]	P value	HR[95% CI]	P value
Age				
<50	Reference		Reference	
≥50	2.01[1.58,2.55]	<0.001	1.22[0.93,1.60]	0.154
Marital status				
Married	Reference		Reference	
Other	1.74[1.43,2.12]	<0.001	1.58[1.23,2.03]	<0.001
Race				
White	Reference		Reference	
Black	1.48[1.16,1.90]	<0.001	1.34[0.97,1.86]	0.077
Other	0.68[0.46,1.00]	0.052	0.65[0.39,1.08]	0.093
Grade				
I	Reference		Reference	
II	1.31[0.91,1.87]	0.143	1.61[0.91,2.86]	0.101
III and IV	2.17[1.53,3.08]	<0.001	4.26[2.47,7.35]	<0.001
Laterality				
Left	Reference		Reference	
Right	0.93[0.77,1.13]	0.476	0.93[0.72,1.19]	0.559
AJCC T stage				
T0/1	Reference		Reference	
T2	1.57[1.22,2.01]	<0.001	1.83[1.31,2.56]	<0.001
T3/4	2.85[2.16,3.76]	<0.001	3.47[2.41,5.01]	<0.001
Subtype				
Luminal A	Reference		Reference	
Luminal B	0.82[0.58,1.15]	0.246	0.85[0.54,1.36]	0.504
HER2 enriched	1.93[1.35,2.75]	<0.001	2.42[1.55,3.78]	<0.001
Triple negative	2.79[2.19,3.56]	<0.001	4.39[3.30,5.85]	<0.001
ER Status				
Positive	Reference		Reference	
Negative	2.70[2.20,3.32]	<0.001	4.17[3.24,5.36]	<0.001
PR Status				
Positive	Reference		Reference	
Negative	2.26[1.86,2.74]	<0.001	3.05[2.37,3.92]	<0.001
HER2 Status				
Positive	Reference		Reference	
Negative	1.07[0.83,1.38]	0.608	1.08[0.78,1.50]	0.647
Radiation				
Yes	Reference		Reference	
No/unknown	1.04[0.84,1.29]	0.689	0.82[0.63,1.06]	0.128
Chemotherapy				
Yes	Reference		Reference	
No/unknown	1.50[1.23,1.82]	<0.001	0.81[0.61,1.07]	0.130
Axillary LN surgery				
SLNB only	Reference		Reference	
SLNB and ALND	1.04[0.84,1.28]	0.728	1.03[0.79,1.35]	0.820

OS, overall survival; BCSS, breast cancer-specific survival; HR, hazard ratio; CI, confidence interval; ER, estrogen receptor; PR, progesterone receptor; HER2, human epidermal growth factor receptor 2; SLNB, sentinel lymph node biopsy; ALND, axillary lymph node dissection.

**Figure 3:**
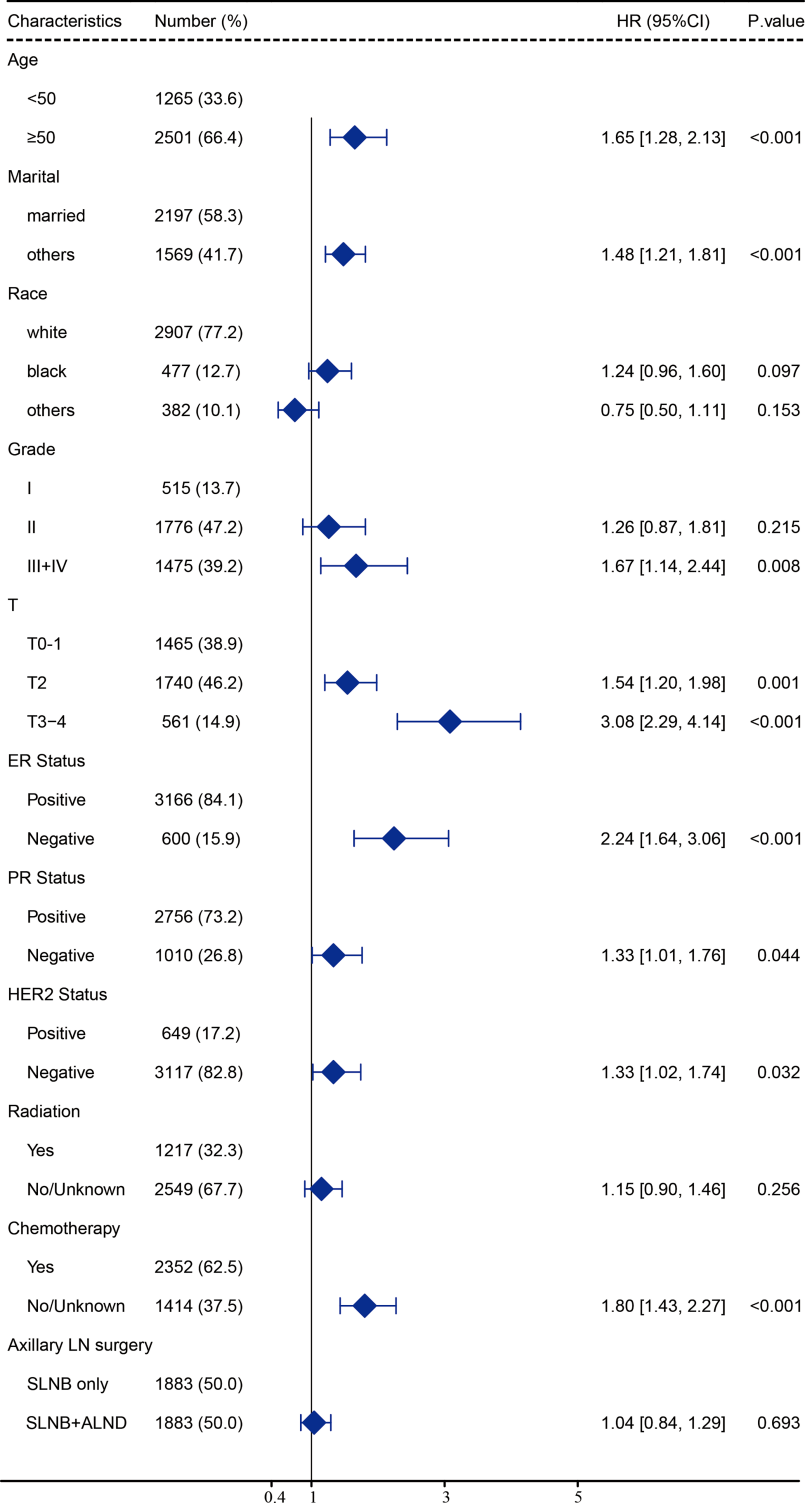
Multivariate Cox regression model forest graph.

After carrying out our univariate analysis, we constructed a multivariate Cox regression model forest graph in order to mine the independent prognostics factors for OS (**Figure 3**). The multivariate analysis results were consistent with the results of the univariate analysis, except for race and HER2 status. As shown in **Figure 3**, the axillary treatment was not statistically significantly associated with OS (HR=1.04, 95% CI: 0.84-1.29, P=0.693) for breast cancer patients, but the adoption of chemotherapy was related with better OS (HR=1.80, 95% CI: 1.43-2.27, P<0.001). In addition, our results indicated that clinicopathological features, such as age, marital status, grade, T stage, ER status, PR status, HER2 status, and chemotherapy were all independent prognostic factors for OS. In **Figure S2**, as for the patients with a single or 2 (macroscopic) metastatic lymph nodes, our results showed that the axillary treatment was not statistically significantly associated with OS (HR=0.95, 95% CI: 0.76-1.17, P=0.606).

### Competing risk model analysis

**Figure 4:**
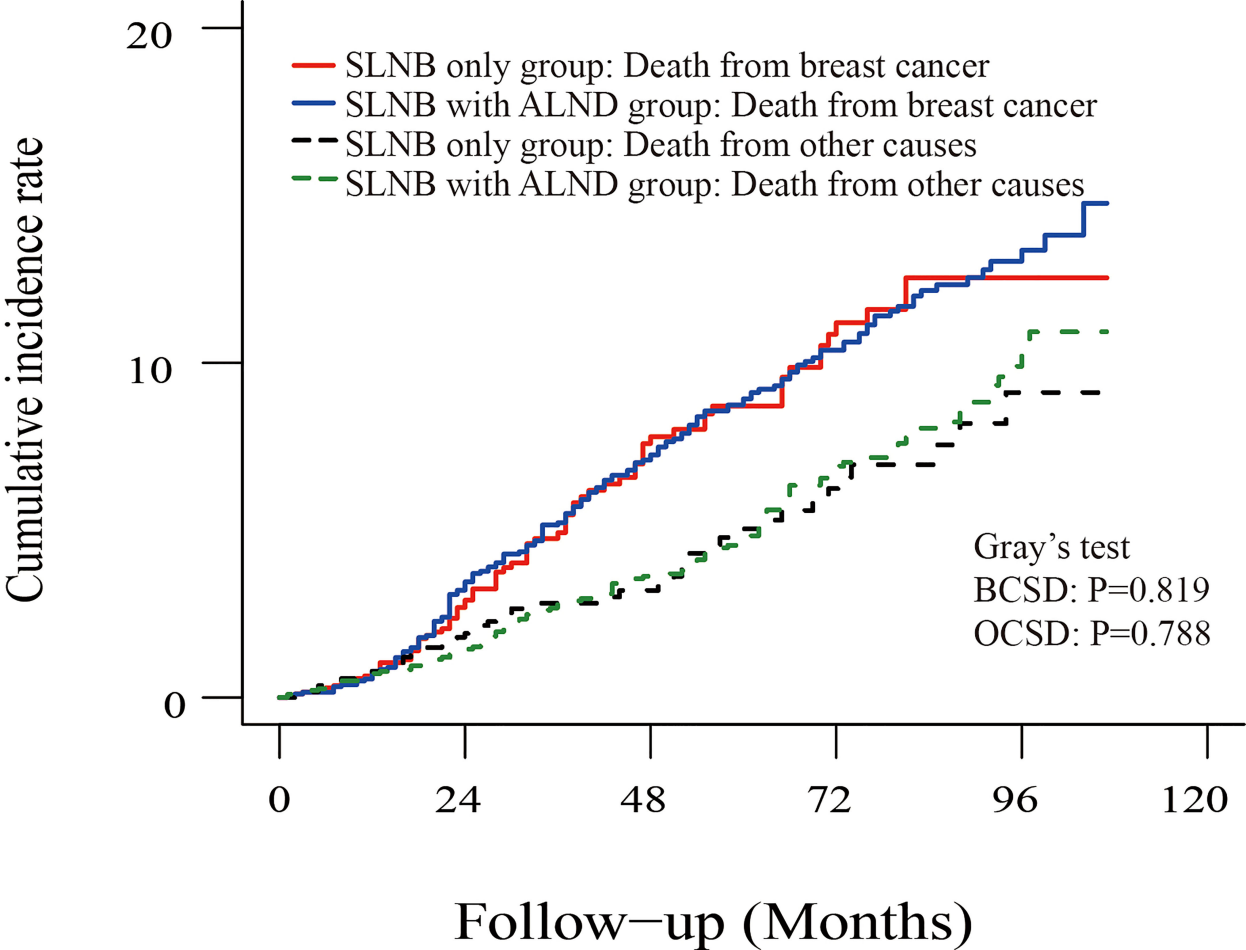
Cumulative incidence of breast-cancer-specific death (BCSD) and other causes of death in the SLNB group and SLNB with complete ALND group.

To reduce the competing risks that could affect the occurrence of BCSD and primary events, we used a competing risk regression model. Compared to the SLNB only group, there was no statistical difference in the cumulative incidence of BCSD (Grey’s test, P=0.819) or OCSD (Grey’s test, P=0.788) for subjects in the SLNB with complete ALND group, as shown in the **Figure 4**. Similarly, there was no difference in the cumulative incidence of BCSD (Grey’s test, P=0.841) or OCSD (Grey’s test, P=0.579) for the patients with a single or 2 (macroscopic) metastatic lymph nodes (**Figure S3**).

### Multivariate competing risk analysis of survival

From our multivariate competing risks regression, we found that marital status and four variables (grade, T stage, ER status and HER2 status) were still independent predictive factors for BCSD (**Table 3**). Additionally, the results once again indicated that axillary treatment was not associated with BCSD (HR=1.13, 95% CI: 0.86-1.48, P=0.400) or OCSD (HR=0.98, 95% CI: 0.70-1.38, P=0.920). Furthermore, patients with Grade I, T0-1 stage, ER, or HER2 positive status, and those who were married tended to have significantly better BCSD than the corresponding group (P<0.05). As for the patients with a single or 2 (macroscopic) metastatic lymph nodes, the results similarly indicated that axillary treatment was not related to BCSD (HR=1.05, 95% CI: 0.80-1.38, P=0.720) or OCSD (HR=0.86, 95% CI: 0.62-1.21, P=0.400) (**Table S3**).

**Table 3:** Multivariate competing risk regression model analysis.

Characteristic	BCSD (N1 = 246, 60.59)		OCSD (N2 = 160, 39.41%)	
	HR[95% CI]	P value	HR[95% CI]	P value
Age				
<50	Reference		Reference	
≥50	1.14[0.85,1.52]	0.380	5.07[2.69,9.56]	<0.001
Marital status				
Married	Reference		Reference	
Other	1.47[1.13,1.91]	0.004	1.41[1.02,1.94]	0.040
Race				
White	Reference		Reference	
Black	0.99[0.69,1.40]	0.940	1.66[1.13,2.43]	0.010
Other	0.66[0.39,1.11]	0.120	0.88[0.47,1.65]	0.690
Grade				
I	Reference		Reference	
II	1.36[0.76,2.44]	0.300	1.20[0.76,1.91]	0.430
III and IV	2.54[1.39,4.64]	0.002	0.92[0.53,1.59]	0.760
AJCC T stage				
T0/1	Reference		Reference	
T2	1.68[1.19,2.36]	0.003	1.34[0.91,1.98]	0.130
T3/4	3.17[2.13,4.72]	<0.001	2.51[1.57,4.01]	<0.001
ER Status				
Positive	Reference		Reference	
Negative	2.60[1.66,4.06]	<0.001	1.22[0.69,2.15]	0.500
PR Status				
Positive	Reference		Reference	
Negative	1.35[0.88,2.07]	0.170	1.25[0.83,1.89]	0.290
HER2 Status				
Positive	Reference		Reference	
Negative	1.64[1.16,2.32]	0.005	0.88[0.58,1.33]	0.540
Radiation				
Yes	Reference		Reference	
No/unknown	1.14[0.85,1.54]	0.380	1.17[0.76,1.80]	0.470
Chemotherapy				
Yes	Reference		Reference	
No/unknown	1.11[0.80,1.54]	0.530	2.98[2.07,4.27]	<0.001
Axillary LN surgery				
SLNB only	Reference		Reference	
SLNB and ALND	1.13[0.86,1.48]	0.400	0.98[0.70,1.38]	0.920

BCSD, breast-cancer-specific death; OCSD, other-cause-specific death; HR, hazard ratio; CI, confidence interval; ER, estrogen receptor; PR, progesterone receptor; HER2, human epidermal growth factor receptor 2; SLNB, sentinel lymph node biopsy; ALND, axillary lymph node dissection.

### Survival analysis of axillary treatment in four molecular subgroups

**Figure 5:**
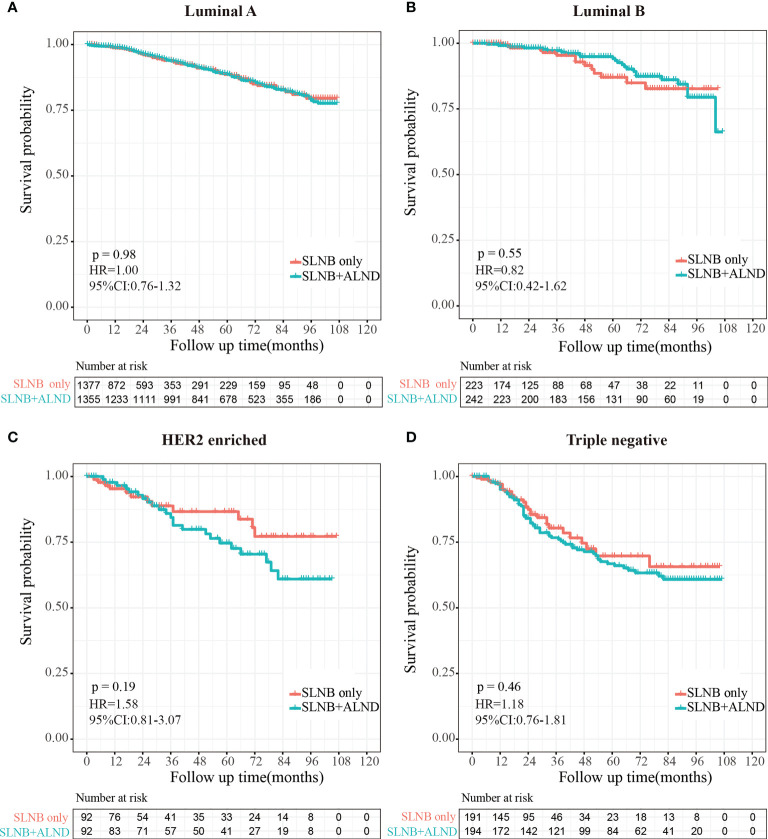
Kaplan-Meier survival analysis for pTxN1miM0 female breast cancer patients in different subgroups. **(A)** Overall survival curves in Luminal A breast cancer between SLNB only group and SLNB with complete ALND group. **(B)** Overall survival curves in Luminal B breast cancer between SLNB only group and SLNB with complete ALND group. **(C)** Overall survival curves in HER2 enriched breast cancer between SLNB only group and SLNB with complete ALND group. **(D)** Overall survival curves in Triple negative breast cancer between SLNB only group and SLNB with complete ALND group.

To investigate the survival prognosis of axillary lymph node treatment in different molecular subgroups further, we performed a Kaplan-Meier analysis. As shown in **Figures 5A, B**, patients in the SLNB with complete ALND group showed no statistical differences from those in the SLNB only group with Luminal A (HR=1.00, 95%CI:0.76-1.32, P=0.98) breast cancer or Luminal B (HR=0.82, 95% CI:0.42-1.62, P=0.55). In addition, and rather unexpectedly, patients in the SLNB only group had similar OS to those in the SLNB with complete ALND group who had HER2-enriched (HR=1.58, 95% CI: 0.81-3.07, P=0.19) or Triple negative (HR=1.18, 95% CI: 0.76-1.81, P=0.46) breast cancers (**Figures 5C, D**).

## Discussion

In this study, based on analysis of a large cohort of 13,848 candidates in the SEER database from 2010 to 2018 and incorporating a range of combined factors into a competing risk regression model, we found that the SLNB with complete ALND group did not show better prognoses than the SLNB only group for female breast cancer patients with SLNB micrometastases. To our knowledge, this is the first study based on a large population to explore the prognostic significance of further axillary lymph node dissection for sentinel lymph node micrometastases in female breast cancer directly through a competing risk model.

Clinicopathological characteristics such as age, TNM stage, tumor grade, and hormone receptor status have been considered reliable prognostic indicators that can be utilized to guide the clinical management of breast cancer patients (30). In our research, some differences in clinicopathological factors were found for almost all essential features in the initial cohort, which could lead to selection bias. Therefore, we implemented PSM analysis to balance these differences. After PSM, the differences that existed in the original cohort no longer emerged, thus allowing our results to objectively reflect the differences between the two groups more accurately. After the PSM procedure, the results of our Kaplan-Meier curve analysis showed that further axillary lymph node dissection did not provide longer OS or BCSS compared to sentinel lymph node biopsy only, and this finding is consistent with many previous papers on breast cancer patients with micrometastases (9, 31–34). According to the results of several clinical trials, further axillary dissection has been abandoned for breast-conserving patients with SLN micrometastases in some centers (31, 35), but it has not been fully analyzed in patients who have undergone mastectomy.

To remove any estimation bias and further investigate the significance of axillary dissection on BCSD and OCSD, we conducted Fine and Gray competing risk model and multivariate competing risk regression analysis. These results showed that there was no statistical difference in the cumulative incidence of BCSD or OCSD for subjects in the SLNB with complete ALND group compared to the SLNB only group. At present, the SLNB technique for lymph node staging has allowed many breast cancer patients to avoid the need for ALND when SLN is found to be negative, thus avoiding many post-operative complications and improving quality of life. Although many experts continue to believe that ALND is mandatory for SLN-positive patients, it is reasonable to question whether it is necessary for all SLN-positive patients to undergo the procedure.

In fact, the question of whether complete ALND is actually required for patients with micrometastasis is an important one because of the long-term prognostic risk of systemic recurrence and local failure associated with residual axillary disease in sentinel lymph node positive patients who elect not to have further axillary surgery, and Controversy still exists surrounding the best management of patients with SLN micrometastases, in terms of further axillary surgery or radiotherapy and/or systemic adjuvant treatment (3, 8, 36). Nevertheless, we were surprised to find that axillary treatment was not an independent factor affecting OS and BCSS, nor was radiotherapy, although many people supposed that ALND and axillary radiotherapy could be alternatives for patients with SLN micrometastases since they may reduce loco-regional recurrence (37).

Importantly, the relative reduction in the number of ALNDs performed over the last decade is supported by a large and growing evidence base (3, 17, 19). In addition, in view of the fact that micrometastases are likely to represent a lower risk of local and distant failure than macrometastases and that women could be spared the morbidity of ALND, at present further intervention for women with SLN micrometastases is primarily surgical treatment, rather than axillary radiotherapy (3, 36). Additionally, since SLN micrometastases are most commonly confirmed following complete pathological assessment, any further axillary surgery needs to take place on a second occasion. Hence, whether radiotherapy can be safely avoided in patients with micrometastases remains unclear, and further studies are still needed to answer this question (36, 38).

After stratifying the patients by characteristics, we found that having an age at diagnosis of 50 years or older, being unmarried, having ER-negative status, PR-negative status, or HER2-negative status, and not receiving systemic chemotherapy were all unfavorable independent factors for the prognosis of patients with micrometastases. Previous studies have also come to the same conclusion, and many studies have highlighted the importance of adjuvant systemic therapy for patients with micrometastases (7, 9, 39, 40). In this study we found that patients in the SLNB with complete ALND group tended to have received chemotherapy. This may be due to the fact that SLN micrometastases could simply represent an indication for systemic adjuvant therapy and patients with SLNB only tended to have a lower tumor burden, or that patients were not willing to tolerate the side effects of chemotherapy.

In the clinical work, breast cancers can be classified into four subtypes based on ER or PR expression and HER2 gene amplification, and the risk and treatment strategies are different for each of these molecular subtypes (41, 42). To investigate the impact of axillary treatment on survival in patients with micrometastases of different molecular subtypes, we performed the subtype analysis. Unfortunately, we found no statistically significant differences in OS between the SLNB only and SLNB with complete ALND groups for any molecular subtype.

Inevitably, this study also has certain limitations. First, as a retrospective study rather than a prospective cohort study, selection bias cannot be ignored and may limit the external effects of this study. Second, the detailed information on family history, comorbidities, endocrine therapy, targeted therapies for HER2, and patients’ underlying health status were unavailable. Finally, longer follow-up times are necessary to obtain more accurate prognostic significance assessments of further axillary dissection for patients with SLN micrometastases.

## Conclusion

In summary, our study examined the prognostic significance of further axillary dissection for sentinel lymph node micrometastases in female breast cancer patients using a large amount of publicly available data. Our results indicate that in early breast cancer patients with micrometastases, complete ALND does not seem to be required and that SLNB suffices to control locoregional and distant disease, with no comparatively adverse effects on survival.

## Data availability statement

The datasets presented in this study can be found in online repositories. The names of the repository/repositories and accession number(s) can be found below: https://seer.cancer.gov.

## Author contributions

YZ and SP drafted the manuscript and analyze data, JS, LD and LS generated the figure, YL performed the background research. NH and YR edited the manuscript. All authors contributed to the article and approved the submitted version.

## Funding

This study was funded by the National Natural Science Fund of China (No.82003183).

## Acknowledgments

We are thankful to the Surveillance, Epidemiology, and End Results Program (National Cancer Institute) for the development of the SEER database. We wish to thank all our colleagues in the Departments of Breast Surgery, First Affiliate Hospital of Xi’an Jiaotong University. The authors also thank AiMi Academic Services (www.aimieditor.com) for the English language editing and review services.

## Conflict of interest

The authors declare that the research was conducted in the absence of any commercial or financial relationships that could be construed as a potential conflict of interest.

## Publisher’s note

All claims expressed in this article are solely those of the authors and do not necessarily represent those of their affiliated organizations, or those of the publisher, the editors and the reviewers. Any product that may be evaluated in this article, or claim that may be made by its manufacturer, is not guaranteed or endorsed by the publisher.

## Supplementary material

Supplementary Figure 1 Kaplan-Meier survival analysis for the cohort of the patients with a single or 2 (macroscopic) metastatic lymph nodes. (A) Overall survival curves in the SLNB only group and SLNB with complete ALND group. (B) Breast cancer-specific survival curves in the SLNB group and SLNB with complete ALND group.

Supplementary Figure 2 Multivariate Cox regression model forest graph in the cohort of the patients with a single or 2 (macroscopic) metastatic lymph nodes.

Supplementary Figure 3 Cumulative incidence of breast-cancer-specific death (BCSD) and other causes of death in the SLNB group and SLNB with complete ALND group in the cohort of the patients with a single or 2 (macroscopic) metastatic lymph nodes.

## References

[1] Lyman GH Giuliano AE Somerfield MR Benson AB 3rd Bodurka DC Burstein HJ. American Society of clinical oncology guideline recommendations for sentinel lymph node biopsy in early-stage breast cancer. J Clin Oncol (2005) 23(30):7703–20. doi: 10.1200/JCO.2005.08.001 16157938

[2] Veronesi U Paganelli G Viale G Luini A Zurrida S Galimberti V. A randomized comparison of sentinel-node biopsy with routine axillary dissection in breast cancer. N Engl J Med (2003) 349(6):546–53. doi: 10.1056/NEJMoa012782 12904519

[3] Yi M Giordano SH Meric-Bernstam F Mittendorf EA Kuerer HM Hwang RF. Trends in and outcomes from sentinel lymph node biopsy (SLNB) alone vs. SLNB with axillary lymph node dissection for node-positive breast cancer patients: experience from the SEER database. Ann Surg Oncol (2010) 17 Suppl 3:343–51. doi: 10.1245/s10434-010-1253-3 PMC4324560 20853057

[4] Chang DW Bressel M Hansen C Blinman P Schofield P Chua BH. Axillary dissection in sentinel lymph node positive breast cancer: Is the staging information worthwhile for patients? Asia Pac J Clin Oncol (2021) 17(2):e27–34. doi: 10.1111/ajco.13238 31461222

[5] Harlow SP Krag DN. Sentinel lymph node–why study it: Implications of the b-32 study. Semin Surg Oncol (2001) 20(3):224–9. doi: 10.1002/ssu.1037 11523107

[6] Harlow SP Krag DN Julian TB Ashikaga T Weaver DL Feldman SA. Prerandomization surgical training for the national surgical adjuvant breast and bowel project (NSABP) b-32 trial: A randomized phase III clinical trial to compare sentinel node resection to conventional axillary dissection in clinically node-negative breast cancer. Ann Surg (2005) 241(1):48–54. doi: 10.1097/01.sla.0000149429.39656.94 15621990 PMC1356845

[7] Patani N Mokbel K. The clinical significance of sentinel lymph node micrometastasis in breast cancer. Breast Cancer Res Treat (2009) 114(3):393–402. doi: 10.1007/s10549-008-0021-6 18425678

[8] Wada N Imoto S. Clinical evidence of breast cancer micrometastasis in the era of sentinel node biopsy. Int J Clin Oncol (2008) 13(1):24–32. doi: 10.1007/s10147-007-0736-0 18307016

[9] Ying-Ying L Tian-Jian Y Guang-Yu L. Prognostic significance of further axillary dissection in breast cancer patients with micrometastases & the number of micrometastases: a SEER population-based analysis. Future Sci OA (2018) 4(5):FSO303. doi: 10.4155/fsoa-2018-0008 29796305 PMC5961405

[10] Iqbal J Ginsburg O Giannakeas V Rochon PA Semple JL Narod SA. The impact of nodal micrometastasis on mortality among women with early-stage breast cancer. Breast Cancer Res Treat (2017) 161(1):103–15. doi: 10.1007/s10549-016-4015-5 27796715

[11] Grube BJ Giuliano AE. Observation of the breast cancer patient with a tumor-positive sentinel node: Implications of the ACOSOG Z0011 trial. Semin Surg Oncol (2001) 20(3):230–7. doi: 10.1002/ssu.1038 11523108

[12] Chu KU Turner RR Hansen NM Brennan MB Bilchik A Giuliano AE. Do all patients with sentinel node metastasis from breast carcinoma need complete axillary node dissection? Ann Surg (1999) 229(4):536–41. doi: 10.1097/00000658-199904000-00013 PMC1191740 10203087

[13] Guenther JM Hansen NM DiFronzo LA Giuliano AE Collins JC Grube BL. Axillary dissection is not required for all patients with breast cancer and positive sentinel nodes. Arch Surg (2003) 138(1):52–6. doi: 10.1001/archsurg.138.1.52 12511150

[14] Meretoja TJ Vironen JH Heikkila PS Leidenius MH. Outcome of selected breast cancer patients with micrometastasis or isolated tumor cells in sentinel node biopsy and no completion axillary lymph node dissection. J Surg Oncol (2010) 102(3):215–9. doi: 10.1002/jso.21608 20740577

[15] Galimberti V Cole BF Viale G Veronesi P Vicini E Intra M. Axillary dissection versus no axillary dissection in patients with breast cancer and sentinel-node micrometastases (IBCSG 23-01): 10-year follow-up of a randomised, controlled phase 3 trial. Lancet Oncol (2018) 19(10):1385–93. doi: 10.1016/S1470-2045(18)30380-2 30196031

[16] Giuliano AE Ballman K McCall L Beitsch P Whitworth PW Blumencranz P. Locoregional recurrence after sentinel lymph node dissection with or without axillary dissection in patients with sentinel lymph node metastases: Long-term follow-up from the American college of surgeons oncology group (Alliance) ACOSOG Z0011 randomized trial. Ann Surg (2016) 264(3):413–20. doi: 10.1097/SLA.0000000000001863 PMC5070540 27513155

[17] Giuliano AE Ballman KV McCall L Beitsch PD Brennan MB Kelemen PR. Effect of axillary dissection vs no axillary dissection on 10-year overall survival among women with invasive breast cancer and sentinel node metastasis: The ACOSOG Z0011 (Alliance) randomized clinical trial. JAMA (2017) 318(10):918–26. doi: 10.1001/jama.2017.11470 PMC5672806 28898379

[18] Dolivet E Loaec C Johnson A Renaudeau C Boiffard F Dravet F. ACOSOG z-0011 criteria impact on axillary surgery for early breast cancer in clinical practice: Evaluation in a retrospective cohort of 1900 patients. Eur J Obstet Gynecol Reprod Biol (2021) 261:41–5. doi: 10.1016/j.ejogrb.2021.04.003 33878635

[19] Bilimoria KY Bentrem DJ Hansen NM Bethke KP Rademaker AW Ko CY. Comparison of sentinel lymph node biopsy alone and completion axillary lymph node dissection for node-positive breast cancer. J Clin Oncol (2009) 27(18):2946–53. doi: 10.1200/JCO.2008.19.5750 19364968

[20] Houvenaeghel G El Hajj H Barrou J Cohen M Raro P De Troyer J. External validation of the SERC trial population: Comparison with the multicenter French cohort, the Swedish and SENOMIC trial populations for breast cancer patients with sentinel node micro-metastasis. Cancers (Basel) (2020) 12(10):2. doi: 10.3390/cancers12102924 PMC7600229 33050650

[21] Andersson Y Bergkvist L Frisell J de Boniface J. Do clinical trials truly mirror their target population? an external validity analysis of national register versus trial data from the Swedish prospective SENOMIC trial on sentinel node micrometastases in breast cancer. Breast Cancer Res Treat (2019) 177(2):469–75. doi: 10.1007/s10549-019-05328-3 PMC6661061 31236811

[22] Gradishar WJ Anderson BO Balassanian R Blair SL Burstein HJ Cyr A. Invasive breast cancer version 1.2016, NCCN clinical practice guidelines in oncology. J Natl Compr Canc Netw (2016) 14(3):324–54. doi: 10.6004/jnccn.2016.0037 26957618

[23] Van Zee KJ Manasseh DM Bevilacqua JL Boolbol SK Fey JV Tan LK. A nomogram for predicting the likelihood of additional nodal metastases in breast cancer patients with a positive sentinel node biopsy. Ann Surg Oncol (2003) 10(10):1140–51. doi: 10.1245/aso.2003.03.015 14654469

[24] Kim T Giuliano AE Lyman GH. Lymphatic mapping and sentinel lymph node biopsy in early-stage breast carcinoma: A metaanalysis. Cancer (2006) 106(1):4–16. doi: 10.1002/cncr.21568 16329134

[25] Moore MP Kinne DW. Axillary lymphadenectomy: a diagnostic and therapeutic procedure. J Surg Oncol (1997) 66(1):2–6. doi: 10.1002/(SICI)1096-9098(199709)66:1<2:AID-JSO2>3.0.CO;2-8 9290685

[26] Li K Wang B Yang Z Yu R Chen H Li Y. Nomogram predicts the role of contralateral prophylactic mastectomy in Male patients with unilateral breast cancer based on SEER database: A competing risk analysis. Front Oncol (2021) 11:587797. doi: 10.3389/fonc.2021.587797 33996535 PMC8117922

[27] Palazzo LL Sheehan DF Tramontano AC Kong CY. Disparities and trends in genetic testing and erlotinib treatment among metastatic non-small cell lung cancer patients. Cancer Epidemiol Biomarkers Prev (2019) 28(5):926–34. doi: 10.1158/1055-9965.EPI-18-0917 PMC6500471 30787053

[28] Shao N Xie C Shi Y Ye R Long J Shi H. Comparison of the 7th and 8th edition of American joint committee on cancer (AJCC) staging systems for breast cancer patients: A surveillance, epidemiology and end results (SEER) analysis. Cancer Manag Res (2019) 11:1433–42. doi: 10.2147/CMAR.S185212 PMC6388984 30863154

[29] Sun W Cheng M Zhou H Huang W Qiu Z. Nomogram predicting cause-specific mortality in nonmetastatic Male breast cancer: A competing risk analysis. J Cancer (2019) 10(3):583–93. doi: 10.7150/jca.28991 PMC6360428 30719155

[30] Sawaki M Yamada A Kumamaru H Miyata H Nakayama K Shimizu C. Clinicopathological characteristics, practical treatments, prognosis, and clinical issues of older breast cancer patients in Japan. Breast Cancer (2021) 28(1):1–8. doi: 10.1007/s12282-020-01188-8 33219915

[31] Galimberti V Cole BF Zurrida S Viale G Luini A Veronesi P. Axillary dissection versus no axillary dissection in patients with sentinel-node micrometastases (IBCSG 23-01): A phase 3 randomised controlled trial. Lancet Oncol (2013) 14(4):297–305. doi: 10.1016/S1470-2045(13)70035-4 23491275 PMC3935346

[32] Galimberti V Botteri E Chifu C Gentilini O Luini A Intra M. Can we avoid axillary dissection in the micrometastatic sentinel node in breast cancer? Breast Cancer Res Treat (2012) 131(3):819–25. doi: 10.1007/s10549-011-1486-2 21468637

[33] Pernas S Gil M Benitez A Bajen MT Climent F Pla MJ. Avoiding axillary treatment in sentinel lymph node micrometastases of breast cancer: a prospective analysis of axillary or distant recurrence. Ann Surg Oncol (2010) 17(3):772–7. doi: 10.1245/s10434-009-0804-y 20183912

[34] Sola M Alberro JA Fraile M Santesteban P Ramos M Fabregas R. Complete axillary lymph node dissection versus clinical follow-up in breast cancer patients with sentinel node micrometastasis: final results from the multicenter clinical trial AATRM 048/13/2000. Ann Surg Oncol (2013) 20(1):120–7. doi: 10.1245/s10434-012-2569-y 22956062

[35] Giuliano AE Hunt KK Ballman KV Beitsch PD Whitworth PW Blumencranz PW. Axillary dissection vs no axillary dissection in women with invasive breast cancer and sentinel node metastasis: A randomized clinical trial. JAMA (2011) 305(6):569–75. doi: 10.1001/jama.2011.90 PMC5389857 21304082

[36] Luo H Yang OO He JL Lan T. Impact of post-mastectomy radiation therapy for sentinel lymph node micrometastases in early-stage breast cancer patients. Med Sci Monit (2022) 28:e933275. doi: 10.12659/MSM.933275 35094003 PMC8812040

[37] Cox CE Kiluk JV Riker AI Cox JM Allred N Ramos DC. Significance of sentinel lymph node micrometastases in human breast cancer. J Am Coll Surg (2008) 206(2):261–8. doi: 10.1016/j.jamcollsurg.2007.08.024 18222378

[38] Wu SP Tam M Shaikh F Lee A Chun J Schnabel F. Post-mastectomy radiation therapy in breast cancer patients with nodal micrometastases. Ann Surg Oncol (2018) 25(9):2620–31. doi: 10.1245/s10434-018-6632-1 29987606

[39] de Boer M van Deurzen CH van Dijck JA Borm GF van Diest PJ Adang EM. Micrometastases or isolated tumor cells and the outcome of breast cancer. N Engl J Med (2009) 361(7):653–63. doi: 10.1056/NEJMoa0904832 19675329

[40] Pepels MJ de Boer M Bult P van Dijck JA van Deurzen CH Menke-Pluymers MB. Regional recurrence in breast cancer patients with sentinel node micrometastases and isolated tumor cells. Ann Surg (2012) 255(1):116–21. doi: 10.1097/SLA.0b013e31823dc616 22183034

[41] Howlader N Cronin KA Kurian AW Andridge R. Differences in breast cancer survival by molecular subtypes in the united states. Cancer Epidemiol Biomarkers Prev (2018) 27(6):619–26. doi: 10.1158/1055-9965.EPI-17-0627 29593010

[42] Waks AG Winer EP. Breast cancer treatment: A review. JAMA (2019) 321(3):288–300. doi: 10.1001/jama.2018.19323 30667505

