# Supplementary figures and images for: The prognostic significance of further axillary dissection for sentinel lymph node micrometastases in female breast cancer: A competing risk analysis using the SEER database
Source: Front Oncol. 2022 Nov 17;12:1012646. doi: 10.3389/fonc.2022.1012646 (PMC9713815; doi:10.3389/fonc.2022.1012646)

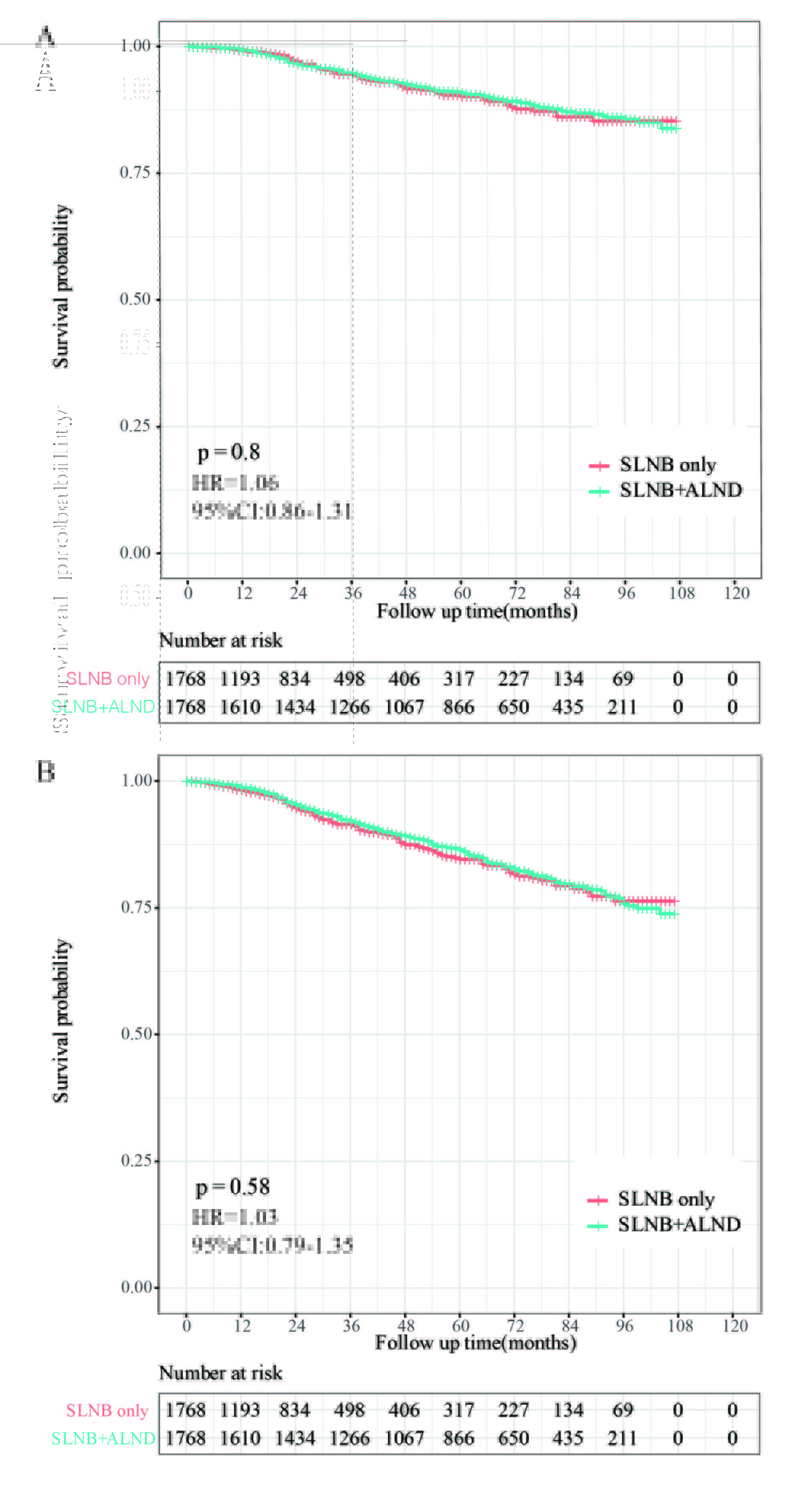

Supplement: Supplementary Figure 1 — Kaplan-Meier survival analysis for the cohort of the patients with a single or 2 (macroscopic) metastatic lymph nodes. (A) Overall survival curves in the SLNB only group and SLNB with complete ALND group. (B) Breast cancer-specific survival curves in the SLNB group and SLNB with complete ALND group. [file Image_1.jpeg]

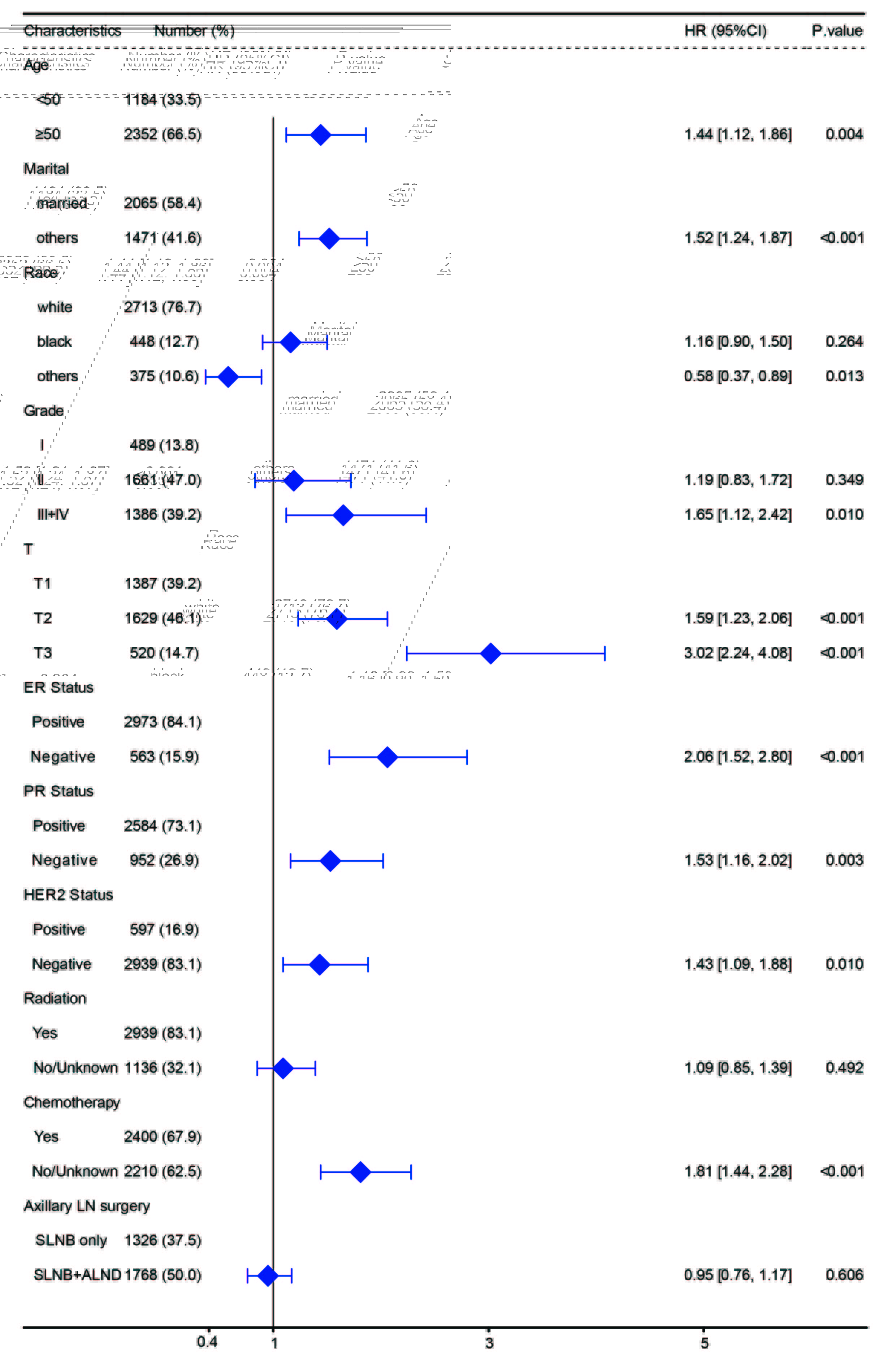

Supplement: Supplementary Figure 2 — Multivariate Cox regression model forest graph in the cohort of the patients with a single or 2 (macroscopic) metastatic lymph nodes. [file Image_2.jpeg]

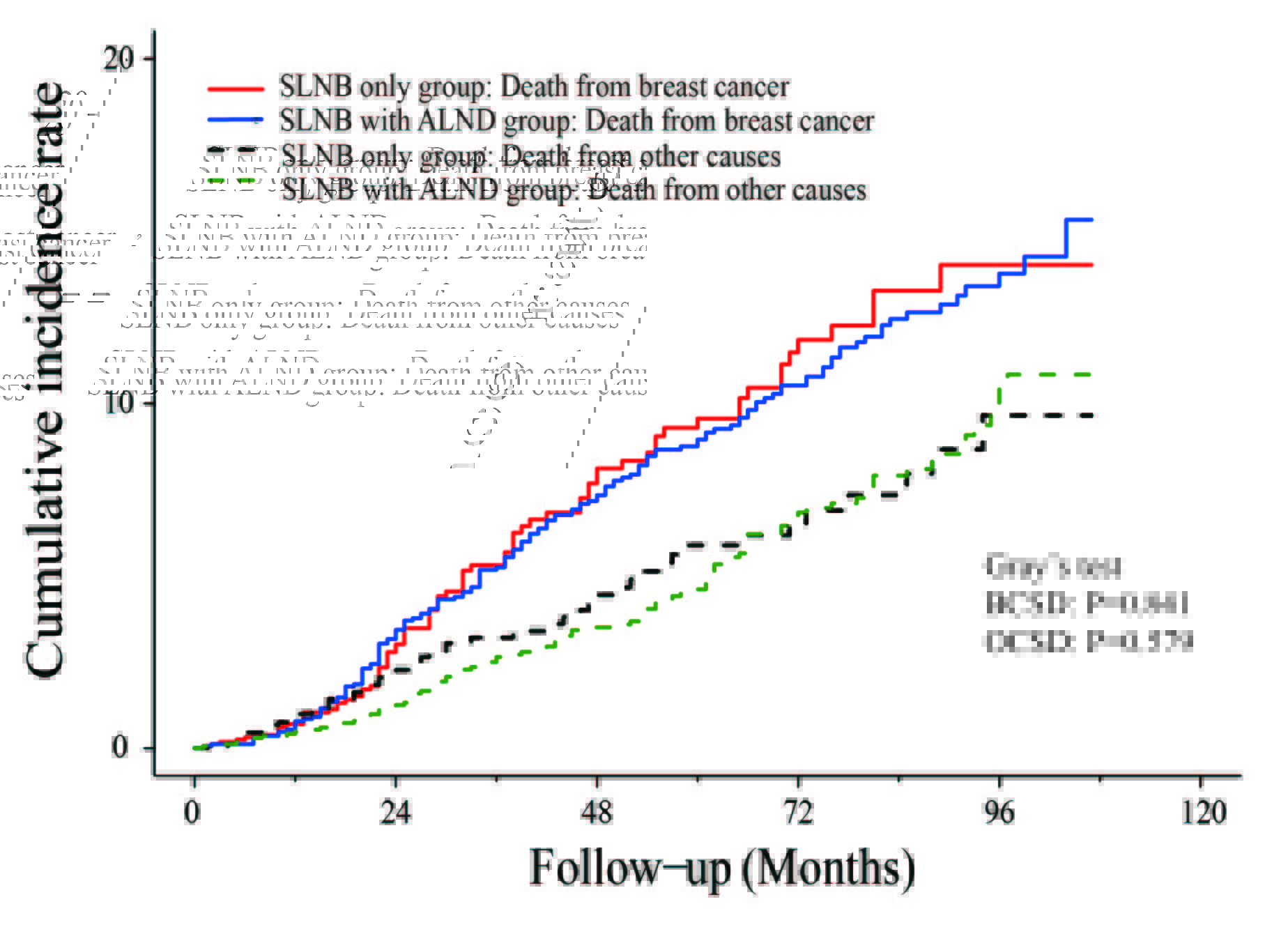

Supplement: Supplementary Figure 3 — Cumulative incidence of breast-cancer-specific death (BCSD) and other causes of death in the SLNB group and SLNB with complete ALND group in the cohort of the patients with a single or 2 (macroscopic) metastatic lymph nodes. [file Image_3.jpeg]
